# Letter to the editor "Dural reconstruction with or without a bone graft of paranasal and anterior skull-base malignancies: Retrospective single-centre analysis of 11 cases and review of literature"

**DOI:** 10.1016/j.bas.2024.102908

**Published:** 2024-08-06

**Authors:** Silambarasan Tamil Selvan

**Affiliations:** Center for Global Health Research, Saveetha Medical College and Hospital, Saveetha Institute of Medical and Technical Sciences, Saveetha University, Chennai, 602105, Tamil Nadu, India

Dear Editor,

I am writing to express my appreciation for the article titled " Dural reconstruction with or without a bone graft of paranasal and anterior skull-base malignancies: Retrospective single-centre analysis of 11 cases and review of the literature" published in the recent issue of Brain and Spine. The results of this research are crucial since they shed light on the efficacy of different reconstruction techniques for complex ASB malignancies, emphasize the importance of multidisciplinary approaches to improving patient outcomes, and emphasize the importance of tailor-made surgical strategies for reducing complications and enhancing survival rates.

This research paper presents critical insight into the surgical management of malignant tumours involving the anterior skull base (ASB), midface, and sinonasal conditions that are both uncommon and challenging. An integrated multidisciplinary approach, including endonasal, endoscopic, and open transcranial/craniofacial techniques, is emphasized to maximize tumour resection and ensure optimal patient outcomes. The intricate anatomy and aggressive growth patterns of these tumours present challenges to surgical intervention, underscoring the need for tailored approaches. Based on the rarity of these cases, the findings from this retrospective study contribute valuable knowledge to the limited literature currently available (Sommer et al., 2023).

This study evaluated outcomes and complications associated with ASB reconstruction, especially with or without bone grafts. The study suggests that structural stabilization with bone grafts may help prevent infections like meningitis and intracranial abscesses caused by cerebrospinal fluid leakage. This research highlights the importance of watertight dural closure and robust structural repair in mitigating these risks. In this study, different reconstruction techniques are compared to contribute to the ongoing debate on surgical methods for repairing ASB defects. A comprehensive account of the morbidity and mortality associated with these complex surgeries is also provided in the paper, including the overall survival and progression-free survival rates of patients. A comprehensive surgical approach combining endoscopic and open techniques performs well in terms of gross total resection (GTR) and disease management, despite its high complication rates. Additionally, this research underscores the necessity of a collaborative, interdisciplinary approach to treating these rare malignancies while also laying the foundation for future studies that aim to improve surgical techniques and patient outcomes (Sommer et al., 2023).

In patients with sinonasal malignancies invading the ASB, this retrospective study assessed outcomes and complications associated with anterior skull base reconstruction. It has been suggested that tabula externa grafts may provide sufficient reinforcement in cases of extensive tumour growth, particularly if it extends into the middle cranial fossa, avoiding complications like cerebrospinal fluid leaks and encephaloceles. There were 46% complications, but 9 out of 11 patients were grossly resected with endoscopic and open surgery. A multidisciplinary approach is crucial to preventing secondary complications, and watertight dural closure is essential. It is nevertheless important to interpret the results cautiously, given the small sample size and the retrospective nature of the study, and there can be no definitive recommendations based on the results (1E).

According to the findings from our retrospective study on the surgical treatment of malignant tumours invading the anterior skull base, midface, and sinonasal cavities, several key directions for future research and practice are evident. The evaluation of multidisciplinary approaches and the impact of adjuvant therapies may optimize postoperative care and improve survival rates while comparing surgical methods and patient selection to different reconstruction techniques can refine surgical methods and patient selection. Through the integration of advanced surgical technologies, we may be able to further improve the precision of tumour resection and reconstruction, which may help reduce complications. Multidisciplinary teams must work collaboratively to create tailored reconstruction approaches based on patient needs and meticulous preoperative imaging and surgical planning. It is necessary to monitor and manage postoperative complications carefully, to identify and treat patients at risk for complications such as CSF leaks, meningitis, and pneumocephalus, and to provide thorough preoperative counselling to mitigate the possibility of these complications occurring.

## Declaration of competing interest

The authors declare that they have no known competing financial interests or personal relationships that could have appeared to influence the work reported in this paper.
